# Discrete SARS-CoV-2 antibody titers track with functional humoral stability

**DOI:** 10.1038/s41467-021-21336-8

**Published:** 2021-02-15

**Authors:** Yannic C. Bartsch, Stephanie Fischinger, Sameed M. Siddiqui, Zhilin Chen, Jingyou Yu, Makda Gebre, Caroline Atyeo, Matthew J. Gorman, Alex Lee Zhu, Jaewon Kang, John S. Burke, Matthew Slein, Matthew J. Gluck, Samuel Beger, Yiyuan Hu, Justin Rhee, Eric Petersen, Benjamin Mormann, Michael de St Aubin, Mohammad A. Hasdianda, Guruprasad Jambaulikar, Edward W. Boyer, Pardis C. Sabeti, Dan H. Barouch, Boris D. Julg, Elon R. Musk, Anil S. Menon, Douglas A. Lauffenburger, Eric J. Nilles, Galit Alter

**Affiliations:** 1 Ragon Institute of MGH, MIT and Harvard, Cambridge, MA USA; 2 Institut für HIV Forschung, Universität Duisburg-Essen, Duisburg, Germany; 3 Computational and Systems Biology Program, Massachusetts Institute of Technology, Cambridge, MA USA; 4 Broad Institute of MIT and Harvard, Cambridge, MA USA; 5 Center for Virology and Vaccine Research, Beth Israel Deaconess Medical Center, Harvard Medical School, Boston, MA USA; 6 Space Exploration Technologies Corp, Hawthorne, CA USA; 7 Icahn School of Medicine at Mount Sinai, Nw York, USA; 8 Harvard Humanitarian Initiative, Cambridge, MA USA; 9 Brigham and Women’s Hospital, Boston, MA USA; 10 Harvard T.H. Chan School of Public Health, Cambridge, MA USA; 11 Howard Hughes Medical Institute, Chevy Chase, MD USA; 12 Massachusetts Consortium on Pandemic Readiness, Cambridge, MA USA; 13 Department of Biological Engineering, Massachusetts Institute of Technology, Cambridge, MA USA

**Keywords:** Antibodies, Viral infection

## Abstract

Antibodies serve as biomarkers of infection, but if sustained can confer long-term immunity. Yet, for most clinically approved vaccines, binding antibody titers only serve as a surrogate of protection. Instead, the ability of vaccine induced antibodies to neutralize or mediate Fc-effector functions is mechanistically linked to protection. While evidence has begun to point to persisting antibody responses among SARS-CoV-2 infected individuals, cases of re-infection have begun to emerge, calling the protective nature of humoral immunity against this highly infectious pathogen into question. Using a community-based surveillance study, we aimed to define the relationship between titers and functional antibody activity to SARS-CoV-2 over time. Here we report significant heterogeneity, but limited decay, across antibody titers amongst 120 identified seroconverters, most of whom had asymptomatic infection. Notably, neutralization, Fc-function, and SARS-CoV-2 specific T cell responses were only observed in subjects that elicited RBD-specific antibody titers above a threshold. The findings point to a switch-like relationship between observed antibody titer and function, where a distinct threshold of activity—defined by the level of antibodies—is required to elicit vigorous humoral and cellular response. This response activity level may be essential for durable protection, potentially explaining why re-infections occur with SARS-CoV-2 and other common coronaviruses.

## Abstract (web summary)

The extent of antibody protection against SARS-CoV-2 remains unclear. Here, using a cohort of 120 seroconverted individuals, the authors longitudinally characterize neutralization, Fc-function, and SARS-CoV-2 specific T cell responses, which they show to be prominent only in those subjects that elicited receptor-binding domain (RBD)-specific antibody titers above a certain threshold, suggesting that development of T cell responses to be related to anti-RBD Ab production.

## Introduction

Following most infections, the persistence of humoral immune responses not only provides a record of infection, but also confers protective immunity upon re-exposure. The emerging data point to sustained humoral immune responses in at least a subset of SARS-CoV-2 infected individuals^1,2^, yet cases of re-infection have begun to emerge^3,4^. Whether persisting antibodies retain neutralizing or other protective antiviral effector functions, remains unclear, but may provide critical clues related to the nature of long-term protection from re-infection. Importantly, higher SARS-CoV-2 antibody levels are consistently observed among severely ill individuals and the elderly, suggesting that enhanced immunity may arise in the presence of more aggressive disease^5^. Nonetheless, the majority of adults experience asymptomatic to mild disease^6^, typically resulting in the generation of lower antibody titers^7^.

Importantly, beyond binding, antibodies confer protection against re-infection or disease via their ability to functionally interfere with infection, either by blocking infection (neutralization) or by recruiting the innate immune system to clear and control disease^8^, both of which have emerged as correlates of immunity against SARS-CoV-2 in vaccine studies in animal models^9,10^. However, the relationship between binding titers and antibody effector function, particularly in individuals with mild-to-asymptomatic disease, is poorly understood. Moreover, how antibody function relates to T cell immunity, proposed as an alternate correlate of immunity, is unclear. Collectively, defining the nature of T and B cell immunity is key to defining the nature of long-lived protection from re-infection.

Here we comprehensively probed the functional humoral immune response in a cohort of 120 seropositive individuals, identified through a community-based prospective seroprevalence study, to gain deeper insights into the spectrum and heterogeneity of functional humoral immunity to SARS-CoV-2 over time. As previously observed, striking heterogeneity in antibody titers were observed across the infected population, positively correlated with the number of symptoms experienced by each individual. Limited antibody waning was noted over the study period, but a discrete titer threshold was observed across the population that discriminated individuals who evolved neutralizing and Fc-effector functions, as well as T cell immunity. These data suggest that a threshold of protective immunity may exist among naturally infected individuals, related to the functional potential of the humoral (and cellular) immune response.

## Results

### Baseline antibody levels track with symptomatology

**Fig. 1:**
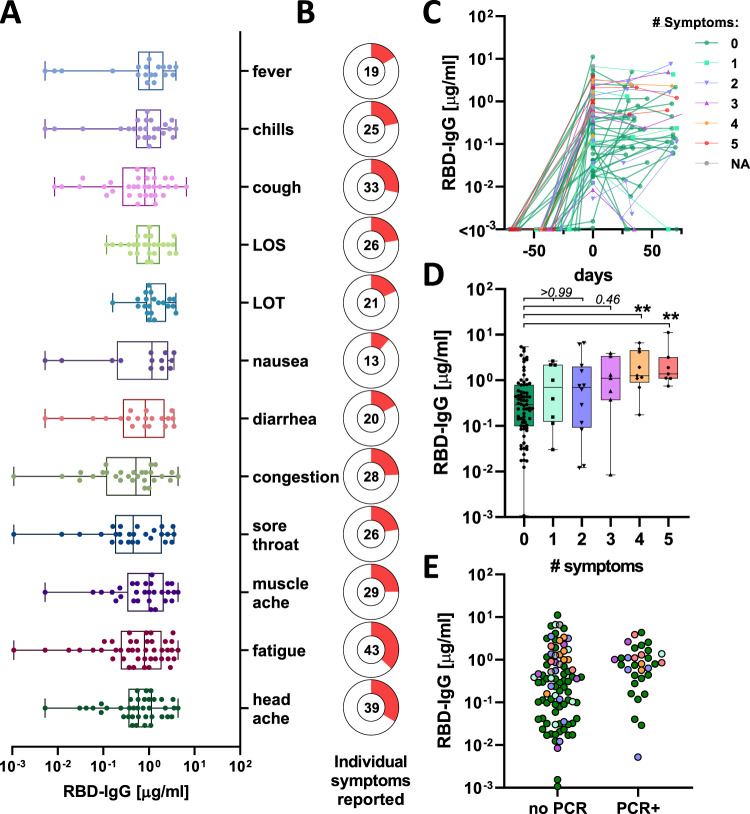
SARS-CoV-2 titer heterogeneity by symptoms. **A** IgG-RBD titer by reported symptom (values for individuals with multiple symptoms are shown for each symptom individually; LOS loss of smell, LOT loss of taste) (box extends from 25th to 75th percentile, whiskers show min and max, and vertical line indicates the median). **B** Donut plots with the proportion of individuals who reported the individual symptom in (**A**), the number in the donut hole indicates the absolute number of individuals (from a total of 116 individuals with symptom data). **C** The line plot shows the trajectory of SARS-CoV-2 RBD-specific antibody titers following seroconversion in 120 individuals (colors and symbols indicate the number of reported symptoms). **D** The whisker box plots show study maximum observed RBD titers grouped by individuals reporting 0–5 symptoms (box extends from 25th to 75th percentile, whiskers show min and max, and the horizontal line indicates the median; *n*_0_ = 73, *n*_1_ = 8, *n*_2_ = 12, *n*_3_ = 7, *n*_4_ = 9, *n*_5_ = 7). **E** The dot plot shows RBD-specific IgG titers in individuals that tested PCR+ (*n* = 32) prior to developing antibody responses or that did not have a PCR test at the time or within 2 weeks prior to seroconversion (*n* = 88; colors indicate the number of reported symptoms as in (**C**) and (**D**)). Statistical differences in (**D**) were assessed with the Kruskal–Wallis test followed by a post hoc Dunn’s correction for multiple testing. Source data are provided as a Source Data file.

In this study we included 4300 volunteers all of whom were employees at Space Exploration Technologies Corp. (SpaceX) that were followed from April 2020, including SARS-CoV-2 receptor-binding domain (RBD) antibody testing, and detailed symptomatology. There were no exclusion criteria and all volunteers were included across all analyses. Following a blinded performance of this SARS-CoV-2 quantitative enzyme-linked immunosorbent assay (ELISA) to the RBD, with a specificity of >99.5%^11^, a total of 120 seroconverters were enrolled. Strikingly, 73 (61%) of the seroconverters reported no COVID-19 related symptoms (including loss of smell, loss of taste, cough, fever, and chills). We observed antibody titers at baseline (T0; first seropositive timepoint) between 1 ng/ml to 11 μg/ml (Fig. 1A and Supplementary Fig. 1). While no single symptom was associated with higher titers, particular symptoms were observed more frequently in the cohort (Fig. 1A, B). Titers were distributed broadly, with a substantial proportion exhibiting levels comparable to subjects who reported multiple COVID-19 related symptoms (fever, chills, cough, loss of smell or loss of taste, Fig. 1C, D). Along these lines, PCR confirmed cases appeared to have higher titers most likely because individuals with COVID-19 related symptoms were more likely to get tested (Fig. 1E). Thus, highly specific SARS-CoV-2 antibodies were readily found in both symptomatic and asymptomatic infection cases, albeit with distributions favoring higher titers in more symptomatic disease.

### Antibody titer kinetics

**Fig. 2:**
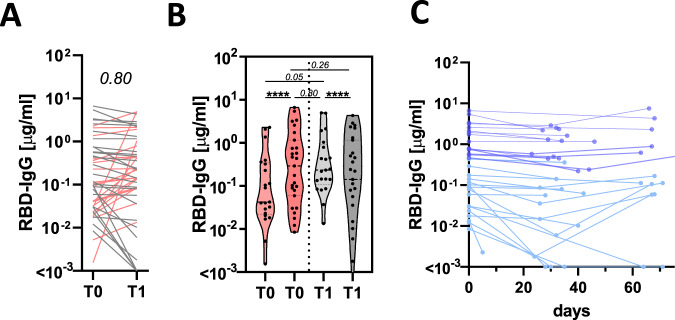
SARS-CoV-2 titer heterogeneity over time. **A** The line graph shows the trajectory of the humoral immune response after the first antibody-positive timepoint (red lines show individuals that experience an increase in their antibody titers and gray shows individuals that exhibit stable or low-level waning) (*n* = 48). **B** The violin plots show the T0 and T1 RBD-specific IgG titers across individuals that experience an increase (reds) or experience stable or decreasing (grays). **C** The line plot shows the overall decay profiles once all samples were aligned based on study maximum observed titer (highest titer per individual observed in this study) (*n* = 32). The shades of blue show the individuals with the higher study maximum titers in the deep blue or lower observed titers in the light blue. Statistical differences between T0 and T1 within a group in (**A**) and (**B**) were assessed with a paired Wilcoxon-test and differences across groups and timepoints were assessed with the Kruskal–Wallis test followed by a post hoc Dunn’s correction for multiple testing. ****p < 0.0001 or exact *p*-values for not significant comparisons. Source data are provided as a Source Data file.

Among the seroconverters, additional longitudinal samples for comprehensive antibody profiling were available for 87 that were included in the study, sampled with a mean interval sampling time of 39.7 days (standard deviation 13.8 days) (Fig. 1C). Forty eight of the seroconverters had at least one additional follow-up test, and 44 (91.6 %) remained seropositive, whereas four individuals lost their antibody responses (Fig. 1C). As RBD IgG titers were evaluated by ELISA over multiple timepoints across the 48 subjects, diverse trajectories were observed, with limited evidence of uniform decay. Twenty-one individuals showed increased titer trajectories at their second timepoint (T1) whereas 27 individuals exhibited lower SARS-CoV-2 IgG levels at T1 (Fig. 2A). The length of timing between sampling timepoints did not appear to influence T0 antibody titers (Supplementary Fig. 1B) or trajectory to the next timepoint (Supplementary Fig. 1C).

The heterogeneous early humoral trajectories were potentially representative of differences in the timing of sampling during the induction of the humoral immune response (Fig. 2B). To test this hypothesis, individuals were grouped based on whether they exhibited increasing or decreasing antibody titers. Subjects with increasing titers tended (*p* = 0.05) to have lower early titers; potentially pointing to a slight upward trajectory from seroconversion to study maximum observed titers, indicating maturation of the response. Conversely, individuals with waning titers exhibited higher titer at the first timepoint (Fig. 2B), pointing to an expected loss of antibodies from study maximum observed immunity, due to a loss of plasmablasts. These data point to an expected rise, peak, and early waning profile observed with other viral infections^12^ and emphasize the need for repeat testing to ascertain the level of waning across a population^13,14^.

Importantly, timepoints were also available for a subset of individuals at additional timepoints. Remarkable stability for up to more than 60 days was observed in these individuals, pointing to a stabilization of the response (Fig. 2C). Although we cannot exclude possible re-exposure and natural boosting of these immune responses, these data argue for some early waning, that stabilizes at later timepoints resulting in persistent seropositivity across this broad titer range. Whether these persisting binding antibodies possess additional antiviral functions, critical for protection against infection/disease, remains unclear.

### Functional implications of titer heterogeneity

Commonly circulating human coronaviruses cause seasonal infections, despite the presence of detectable antibody levels across the population^15,16^. However, why some individuals continue to get re-infected, despite the presence of antibody-titers is unclear. Beyond binding, the ability of antibodies to neutralize and leverage innate immune effector functions is key to protection across many clinically approved vaccines^8^ as well as against SARS-CoV-2 in animal models^9,10^. Whereas some literature points to a relationship between RBD-binding titers and neutralization^17^, the overall relationship between binding and humoral function is not well established.

**Fig. 3:**
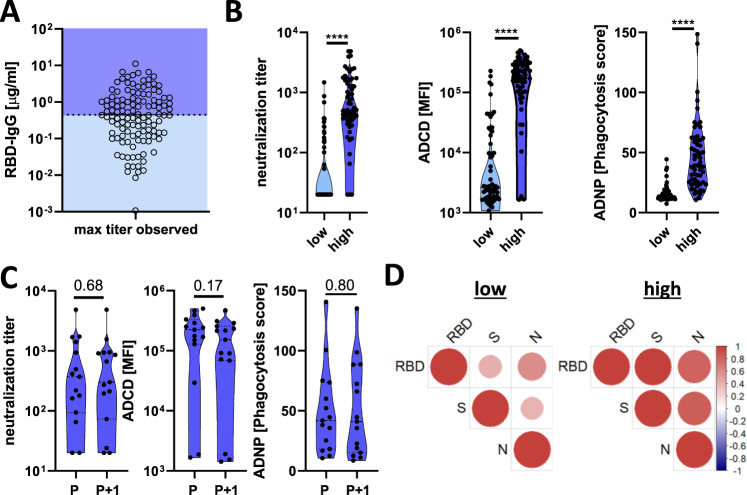
A discrete titer cut-off tracks with functional SARS-CoV-2 humoral and T cell immunity. **A** The dot plot shows the distribution of study maximum observed antibody titers (highest titer per individual observed in this study) across the cohort, split based on median titers. Dark blue shading indicates all individuals above the median and the light blue shows all the individuals below the median (*n*_total _= 120; *n*_low_ = 60, *n*_high_ = 60). **B** The violin plots show the distribution of neutralizing antibody titers (dilution factor, left), antibody-dependent complement deposition (ADCD, mean fluorescence intensity, middle), and antibody-dependent neutrophil phagocytosis (ADNP, phagocytosis score, right)(*n*_low_ = 60, *n*_high_ = 60) against SARS-CoV-2 S. **C** The violin plots show the neutralization levels, ADCD, and ADNP (*n* = 15) from the maximum observed titers to the next timepoint (*P* + 1) in a subset of individuals in the high titer group. **D** The correlation heat maps (Spearman-correlation) show significant correlations (*p* < 0.05) between RBD, Spike (S), and Nucleocapsid (N) titers in the high titer (right; *n* = 15) and low titer (left; *n* = 26) groups. Statistical differences between two groups were assessed with a two-sided non-parametric Mann–Whitney test in (**B**) and paired Wilcoxon-test in (**C**). ****p < 0.0001 or exact *p*-values for not significant comparisons. Source data are provided as a Source Data file.

To begin to address the relationship between antibody titer and function, the 120 seropositive individuals were split in a simple unbiased manner by the median study maximum observed titers and examined through multiple functional assays (Fig. 3A). Remarkable differences in antibody effector function were observed across the median-split (Fig. 3B): detectable neutralization, antibody-mediated complement deposition (ADCD), and antibody-dependent neutrophil phagocytosis (ADNP) were observed almost exclusively in individuals possessing higher maximum observed RBD-specific antibody titers. Furthermore, these functions remained stable in individuals with repeat timepoints (Fig. 3C).

**Fig. 4:**
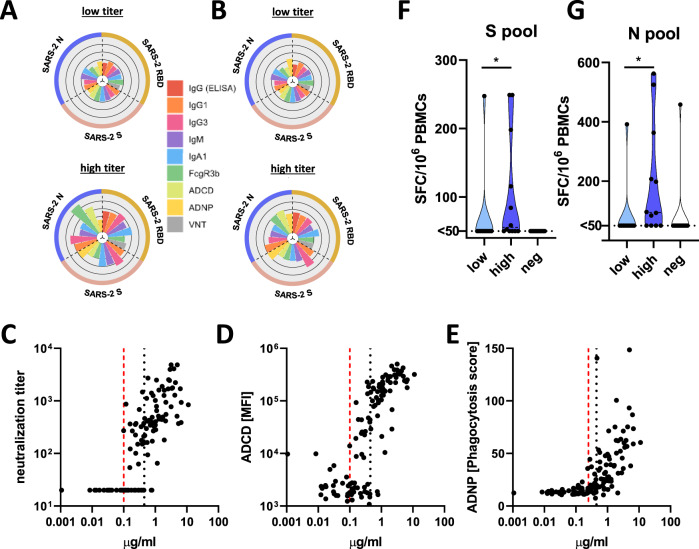
A discrete titer cut-off tracks with functional SARS-CoV-2 humoral and T cell immunity. **A**, **B** The flower plots summarize titer and functional data against SARS-CoV-2 RBD, S or N antigen in high or low titer group at maximum observed titers (**A**) and the following timepoint (**B**) (the petal color corresponds to features as indicated; the univariate data are also shown in Fig. 3 and Supplementary Fig. 2). **C**–**E** Antibody-dependent virus neutralization (**C**), ADCD (**D**) or ADNP (**E**) by ELISA RBD titer. The black dotted line indicates the median split (0.45 μg/ml) and the red dashed line the threshold titer for the individual functions. **F**, **G** The violin plots show the number of spots forming cells (SFC) of interferon-gamma (IFNγ) secreting T cells after overnight stimulation with either an overlapping peptide pool covering SARS-CoV-2 S (**F**) or N (**G**) in individuals with low titers (light blue, *n* = 10), high titers (dark blue, *n* = 12) or negative controls (white, *n* = 14). Statistical differences between two groups were assessed with a two-sided non-parametric Mann–Whitney test (in (**F**) *:*p* = 0.039, in (**G**) *:*p* = 0.018). Source data are provided as a Source Data file.

We next probed the functional humoral profile across additional SARS-CoV-2 antigens, aimed at defining whether additional humoral specificities may compensate for poor RBD-functional immunity. Consistent coordination of humoral titers across RBD, nucleocapsid (N), and full spike (S) were observed (Fig. 3D), albeit the correlations were stronger in the high titer group. Accordingly, despite the correlated nature of RBD-, N-, and S-humoral responses across both groups, only individuals with high IgG titers exhibited broad and robust RBD-, N-, and S-humoral immune responses of different subclasses, isotypes, and with additional innate immune effector functions both at the first (Fig. 4A, Supplementary Fig. 2) and second timepoints (Fig. 4B). In contrast, limited humoral immune responses across all 3 antigens were observed in individuals with low RBD-titers. Beyond the median split (0.45 μg/ml), more discrete titers were noted that tracked with individual antibody functions, where neutralization emerged at a cut off of 0.1 μg/ml, ADNP only appeared at 0.25 μg/ml, and complement appeared in some individuals at concentrations of IgG as low as 0.1 μg/ml (Fig. 4C–E). These differences may reflect the distinct numbers of antibody molecules required to drive each function, representing unique functional needs to cross-link viral spikes, Fc-receptors, or deposit complement, respectively. Yet these distinct thresholds point to opportunities to define discrete titer thresholds for each antibody effector function that may ultimately define quantitative antibody functional correlates of immunity in future large-scale sero-surveillance cohorts. Together, these data indicate that antibodies to other SARS-CoV-2 antigen specificities may not compensate for low RBD-specific functional antibody levels. Rather, it appears that a distinct titer threshold may track with durable functional humoral immune responses to RBD and other SARS-CoV-2 antigens.

The absence of high binding, neutralization, or antibody effector function does not ultimately rule out protection from re-infection, and speculation has emerged about the potential role of T cells, rather than antibodies, as critical correlates of immunity in COVID-19, particularly in asymptomatic/mild disease^18^. Thus, given the emerging data pointing to the presence of T cell immunity both in infected and uninfected populations^18,19^, we next assessed the presence of SARS-CoV-2 specific T cell responses in our cohort. Following T-cell expansion culture, responses to either SARS-CoV-2 spike protein (S) or nucleocapsids protein (N) overlapping peptide pools were quantified by IFNγ ELISpot in 12 high and 10 low RBD antibody titer individuals.

We observed SARS-CoV-2 specific T cells in 83 % (10 of 12) of the individuals in the high titer group against at least one of the tested peptide pools, and only 10% (1 of 10) of the individuals in the low titer group had detectable T cell reactivity against the S and N pools (Fig. 4F, G). Conversely, S- and N-specific T cells were readily detectable in hospitalized SARS-CoV-2 infected individuals or symptomatic convalescent individuals (Supplementary Fig. 3), while only 1 of 14 seronegative and 1 of the 16 pre-pandemic controls also possessed presumably cross-reactive T cells^18^. Finally, while individuals with asymptomatic infection harbored low T cell numbers, a non-significant inverse trend was observed between symptom and T cell numbers, pointing to a potential role for T cells in disease attenuation (Supplementary Fig. 3). These findings demonstrate that SARS-CoV-2 specific T cells are not detectable in all infected individuals, and are not selectively enhanced among individuals with less robust humoral immune responses. Instead, the data suggest that both T and B cells evolve in a coordinated manner. A discrete titer cut off marked the generation of persistent diverse functional humoral and cellular immune responses in a subset of SARS-CoV-2 infected individuals that may collectively contribute to protection upon re-exposure.

## Discussion

The recent SARS-CoV-2 pandemic has left parts of the world paralyzed. Our lack of understanding the predictors of disease severity has overwhelmed our hospital systems. Waning antibody titers^7,14^ and new cases of re-infection suggest that immunity may only be transient and incomplete and new emerging viral variants clearly have begun to escape natural immunity^4,20^. However, for many pathogens and vaccines, specific antibody levels or functions represent the critical protective threshold of immunity^21^. While emerging data have begun to show that antibodies represent vital biomarkers that capture infection rates more comprehensively than nucleic acid-based testing^13^, the precise levels of antibodies associated with protection from re-infection remain unclear. Probing the evolution of the humoral immune responses in a community-based serosurveillance study, here we observed that while the SARS-CoV-2 specific humoral immune response is largely stable for several months, the presence of antibodies does not automatically track with sustained functional cellular or humoral immunity to SARS-CoV-2 that may be required for long-term protection against re-infection. Coupled to large re-infection serosurveillance cohorts, able to also capture viral loads and inflammatory status, the precise cut-off of this titer may be ascertained and used to guide vaccine prioritization.

Antibody titers have been linked intimately to disease severity^5^, leading some to argue that antibodies are less critical for disease control. However, emerging data point to the functional quality, rather than the quantity of the humoral immune response, as a correlate of immunity^22,23^. For example, vaccine-induced antibodies lacking the ability to recruit NK cells or monocytes fail to protect against malaria challenge^24^. While previous studies have noted a robust correlation between RBD-specific antibodies and neutralization^17^, not all antibodies against the RBD are neutralizing^25^. Furthermore, neutralization also accrues with SARS-CoV-2 disease severity^26^. Thus, low titer antibodies emerging following asymptomatic or mild disease may not necessarily possess the key footprints required to block viral infection. Likewise, innate immune recruiting antibodies were also only observed at titers above 0.1 μg/ml (Fig. 4), likely due to the requirement of sufficient antibodies to form immune complexes that cluster Fc-receptors and drive cellular activation^27^. Thus, a minimal titer may mark the evolution of a sufficiently broad neutralization footprint and the generation of sufficient antibodies to recruit antibody effector function.

The immune decision to generate a robust or weak humoral immune response may occur at the time of the host-pathogen interaction, dependent on the level of viral challenge, or inflammatory cues. Low-level challenge may elicit only weak, poorly functional antibodies. Conversely, high-burden challenge may lead to the generation of a potent and functionally robust humoral immune response, programmed to respond aggressively upon re-encounter with the pathogen. The immune decision may also occur at the level of host genetics or gender, where human leukocyte-antigens (HLA) alleles and sex have been clearly linked with differential response to vaccination^28,29^. While we were underpowered to probe these differences, and lacked qPCR viral load levels early in the pandemic, future large-scale re-infection studies will have the potential to probe the demographic, inflammatory, and viral modulators of immunity to SARS-CoV-2, beyond the force of exposure or symptomatology.

The presence of cross-reactive T cell immunity across both pre-pandemic and otherwise healthy individuals have raised the possibility that antibodies developed against endemic common coronaviruses may confer complementary or compensatory immunity in individuals that experience asymptomatic or mild infection^30^. However, using a highly sensitive T cell expansion analysis, SARS-CoV-2 specific T cell responses were solely observed in individuals that elicited broad functional humoral immune responses. These data point to limited evidence for a compensatory T cell signature in asymptomatic/mild disease. Conversely, given that robust T cell immune responses were observed in convalescent subjects with symptomatic infection, these data suggest that T and B cell responses likely evolve synchronously, driven by symptomatic infection (Supplementary Fig. 3). However, whether additional tissue-resident cells may exist and persist in the respiratory tract of asymptomatic individuals that generate lower antibody responses remains unclear.

Unlike natural asymptomatic/mild infection, SARS-CoV-2 vaccines appear to drive robust humoral immune responses, nearly all eliciting neutralization at levels observed in symptomatic convalescents after two rounds of immunization^31–34^ and some able to drive the co-evolution of Fc-effector function^10^, both linked to protection from SARS-CoV-2 challenge^20^. The need for multiple rounds of immunization suggests that more antigen or boosting may be required to push the immune system to generate functional immunity that may be required for protection. Thus, vaccine boosting, unlike mild/asymptomatic natural infection, is likely to result in the induction of broad robust protective immunity. Moreover, several vaccine platforms also induce T cell immunity, which may not be necessary for vaccine-induced sterilizing protection but may cooperate with antibodies to drive control and clearance should infection occur. The data presented here point to a critical functional immunologic threshold—simply captured at the level of antibody titers—that may exist in natural infection, that may guide surveillance efforts and provide insights for the prioritization of vaccine campaign efforts to immunize those most vulnerable to re-infection.

## Methods

### Cohort

The parent cohort study was launched in mid-April 2020, providing an opportunity for industry employees to volunteer for COVID-19 testing and surveillance (Space Exploration Technologies Corp.). All employees were invited to participate by email. There were no exclusion criteria. Upon obtaining informed consent, blood samples were collected approximately every 39.7 days (standard deviation 13.8 days) and participants completed a study survey at the initiation of the study and thereafter, including the collection of COVID-19 related symptoms. The median age of the seropositive population was 31 years (range 22–71 years) and 92% were males with an average BMI of 28.0 kg/m^2^ (range 18.5–42.4 kg/m^2^) resembling the characteristics of the parent cohort (median age: 32 years, range: 18–71 years, 84.3% male (3582/4245 individuals with reported gender) and BMI of 27.0 kg/m^2^ (range 15.7–60.9 kg/m^2^). The study protocol was approved by the Western Institutional Review Board. The use of de-identified data and biological samples was approved by the Mass General Brigham Healthcare (previously Partners Healthcare) Institutional Review Board. All participants provided written informed consent.

### RBD-IgG ELISA

Serological analyses were performed using an in-house ELISA that detects IgG against the RBD of the SARS-CoV-2 spike glycoprotein (provided by Aaron Schmidt) using a previously described method^35^. The assay was further evaluated in a blinded proficiency study against several EUA approved ELISAs, demonstrating >99.5% specificity^11,35^. Briefly, 384-well plates were coated with 0.5 μg/ml of RBD for 1 h at 37 °C in bi-carbonate buffer. The plates were then washed and plasma samples were added at a 1:100 dilution in duplicate for 1 h at 37 °C, washed and then detected with a secondary anti-human-IgG-HRP (Bethyl Laboratories). The secondary was washed away after 1 h, and the colorimetric detector was added (TMB; Thermo Fisher) for 5 min, the reaction was stopped and the absorbance was acquired at 450/570 nm on a Tecan infinite M1000pro plate reader and Tecan-i-control V.3.4.2 software (Biotek Instruments). In order to convert raw OD values into concentration (μg/ml) a 12 two-fold dilution curve (starting at 625 ng/ml) of an SARS-CoV-2 RBD-specific monoclonal IgG1 (clone: CR3022) was included onto every ELISA plate. The sample concentration was interpolated from the resulting standard curve, as previously described^35^. A positive cutoff was equal to the mean of the OD-converted μg/ml values of the negative control wells on the respective plate plus five times the standard deviation of the concentration from negative plasma samples.

### Antigen biotinylation

For all antibody-based assays, SARS2-CoV2-nucleocapsid (N) (Aalto Bio Reagents Ltd) and SARS2-CoV2-Spike (S) (provided by Eric Fisher) antigen were biotinylated using Sulfo-NHS LCLC biotin (Thermo Fisher) and excessive biotin removed with ZebaSpin desalting columns (7 KDa cut-off, Thermo Fisher).

### IgG subclass, isotype, and FcγR binding

SARS-CoV-2 specific antibody subclass and isotypes, and FcγR binding was analyzed using a custom Luminex multiplexed assay^36^. SARS2-CoV2-RBD, SARS2-CoV2-N, and SARS2-CoV2-S were coupled to magnetic Luminex beads (Luminex Corp, TX, USA) by carbodiimide-NHS ester-coupling (Thermo Fisher). Dilution curves were performed on pooled samples from the cohort to determine dilutions in the linear range for each detection reagent. Coupled beads were then incubated with different plasma dilutions (between 1:100 and 1:1000 depending on the secondary reagent) for 2 h at room temperature in 384-well plates (Greiner Bio-One, Germany). Unbound antibodies were washed away and PE-conjugated antibody (Southern Biotech, AL, USA, see Life Science Reporting Summary) at a 1:100 dilution used to detect IgG1 (#9054-09), IgG3 (#9210-09), IgM (#9020-09) or IgA1 (#9130-09), respectively. For the FcγR3b binding, a PE-Streptavidin (Agilent Technologies, CA, USA) coupled recombinant biotinylated human FcγR3b protein (Duke Protein Production Facility) was used as a secondary probe. After 1h incubation, the excessive secondary reagent was washed away and the relative antibody concentration per antigen determined on an IQue analyzer (IntelliCyt, NM, USA). Samples with mean signals plus five times the standard deviation of the PBS-control wells were considered as positive.

### ADCD

Antibody-Dependent-Complement-Deposition was assessed as described previously^37^. In brief, biotinylated antigens were coupled to red fluorescent Neutravidin beads (Thermo Fisher). Plasma antibodies were diluted 1:10 in 0.1% BSA and incubated with the coupled antigen beads for 2 h at 37 °C. Beads were washed with PBS and incubated with complement factors from guinea pig (Cedarlane) at a 1:100 dilution in Gelatin Veronal Buffer (with Mg & Ca) (Boston BioProducts) for 20 min at 37 °C. The complement reaction was then stopped by washing with 15 mM EDTA in PBS. C3 deposition on the beads was detected with a 1:100 dilution of a FITC conjugated anti-guinea pig C3 antibody (MP Biomedical, # 0855385) and relative C3 deposition was analyzed on an iQue analyzer.

### ADNP

Antibody-Dependent-Neutrophil-Phagocytosis was analyzed as described previously^38^. Briefly, biotinylated antigens were coupled to green fluorescent Neutravidin beads (Thermo Fisher) and immune complexes were formed by incubating a 1:100 plasma dilution with the beads for 2 h at 37 °C in 96-well plates (Greiner Bio-One). Human neutrophils were obtained from ACK lysed blood from healthy donor whole blood and 2 × 10^5^ cells were incubated with the formed immune complexes. After 1 h at 37 °C, cells were washed, and surface stained with an anti-human CD66b antibody (Biolegend, #305112) at a 1:100 dilution. Neutrophil phagocytosis was analyzed on an iQue flow cytometer and a phagocytosis score was calculated as the product of the frequency of bead positive CD66b neutrophils and the mean fluorescence of the bead positive cells using ForeCyt Standard Edition 8.1 software (Supplementary Fig. 4).

### SARS-CoV2 antibody-mediated virus neutralization

The ability of antibodies to neutralize the virus was assessed on a 2019-nCoV pseudovirus neutralization assay, as described previously^39^. In brief, HEK293T cells were transfected with pcDNA3.1(-)-hACE2 (Addgene). 12 h post transfection; the HEK293T/hACE2 cells were seeded in 96-well plates (2 × 10^4^ cells/well) and incubated overnight. Heat (56 °C, 30 min) inactivated plasma samples were serially diluted and mixed with 50 μl of pseudoviruses, incubated at 37 °C for 1 h and added to the HEK293T/hACE2 cells. Forty-eight hours after infection, cells were lysed in Steady-Glo Luciferase Assay detection (Promega). A standard quantity of cell lysate was used in the luciferase assay with luciferase assay reagent (Promega) according to the manufacturer’s protocol.

### PBMC isolation and T cell expansion

PBMCs were isolated and frozen from EDTA blood within 24 h after collection using Sepmate tubes (Stemcell Technology). Before the ELISPOT assay, PBMC samples were thawed and cultured in R10-50 media (RPMI media supplemented with 10% FCS, penicillin/streptavidin, 2 mM L-Glutamine, 10 mM HEPES buffer, and 50 U/ml IL-2) containing 0.1 μg/ml anti-human CD3 (clone: 12F6, Absolute Antibody Ltd., #AB00640-2.0). Cells were inspected daily and R10-50 (w/o anti-CD3) media was added/replaced as needed. After 8 days, R10-50 media was replaced with R10 media (no IL-2 or anti-CD3) and cells were rested overnight.

### ELISPOT

PVDV membrane plates (Millipore, MA, USA) were coated with anti-human IFNγ antibody (clone: 1-DK1, Mabtech Inc, #3420-3-1000, conc.: 2 μg/ml) overnight. Expanded and overnight rested PBMC samples (see above) were counted and 3 × 10^5^ PBMCs were added per well with S or N overlapping peptide pools (both Miltenyi, Germany) at 1.25 μg/ml peptide, and incubated overnight. Medium alone was used as a negative control. Pools of 23 MHC-I restricted peptides from human Cytomegalovirus, Eppstein Barr virus and Influenza virus (CEF, Anaspec Inc.) and 35 MHC-II restricted peptides from human Cytomegalovirus, Epstein Barr virus, Influenza virus, Tetanus toxin, and Adenovirus 5 (CEFTA, Mabtech Inc.) were used as positive controls. IFNγ secretion was detected with a biotinylated anti-human IFNγ antibody (clone: 7-B6-1, Mabtech Inc, #3420-6-1000, conc. 0.5 μg/ml) and ALP conjugated-Streptavidin (Mabtech Inc). Spots were developed with 1-Step BCIP/NBT-plus reagent (Mabtech Inc.) for 20 min. Membranes were dried and spots were analyzed and counted on an ImmunoSpot CTL analyzer. A response was considered positive only if there were ≥50 SFCs/10^6^ PBMC after subtracting the value of the matched negative control.

### Statistics

Microsoft Excel 365 was used to compile experimental data and patient information. Violin plots, bar graphs, and *x-y* plots were generated in Graph Pad Prism V.8. Statistical differences between two groups were calculated using a two-sided Mann–Whitney test or Wilcoxon test for paired comparisons. To compare multiple groups, a Kruskal–Wallis test was used followed by the Dunn’s method correcting for multiple comparisons in Graph Pad Prism V.8 (significance levels: *p < 0.05, **p < 0.01, ***p < 0.001, ****p ≤ 0.0001). Flower plots were visualized with the ggplot2 package (v.3.3) in R (v.4.0.1) and RStudio (v.1.3) using Z-scored values.

### Reporting summary

Further information on research design is available in the Nature Research Reporting Summary linked to this article.

## Supplementary information

Supplementary Information

Reporting Summary

## Data Availability

All relevant data are included in this manuscript. No data was stored externally. Source data are provided with this paper.

## Source data

Source Data

## Code availability

There was no specific custom code used in this manuscript.
