# Supplementary material for: Discrete SARS-CoV-2 antibody titers track with functional humoral stability
Source: Nat Commun. 2021 Feb 15;12:1018. doi: 10.1038/s41467-021-21336-8 (PMC7884400; doi:10.1038/s41467-021-21336-8)
Supplement: Supplementary file 1 — Supplementary Information [file 41467_2021_21336_MOESM1_ESM.pdf]

Supplementary Information

Discrete SARS-CoV-2 antibody titers track with functional humoral stability

Bartsch et.al

Supplementary Figure 1-4

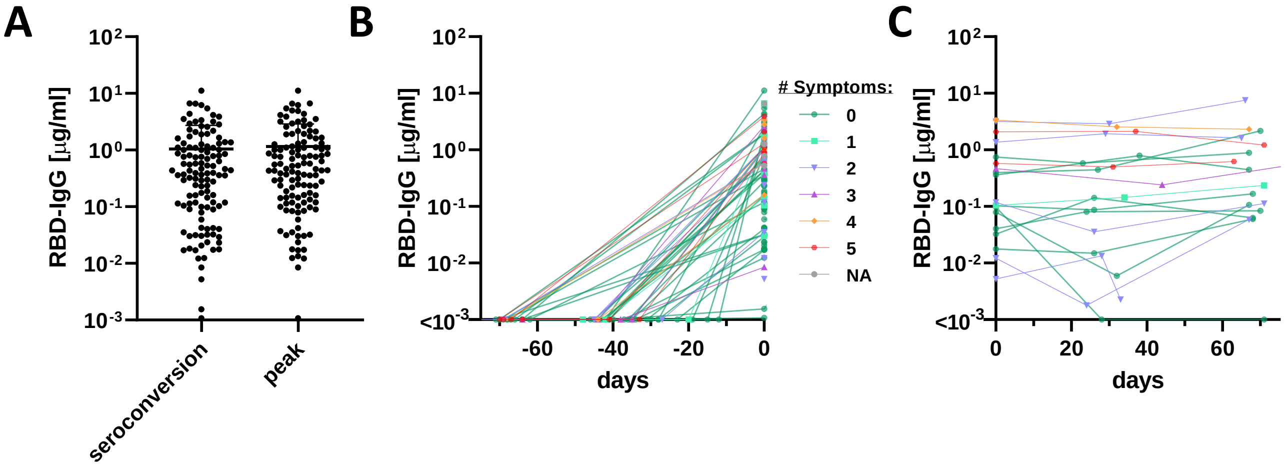

Supplementary Figure 1. Antibody profiles broken up over time. (A) The dot plot shows RBD-specific antibody titers at seroconversion and then at maximum observed immunogenicity for the cohort (n =120, error bar represents the standard deviation). (B) The line plot shows the timing of antibody increase from last seronegative to first seropositive (n=45). (C) The line plot shows the rates of antibody decline over several time points (n=19). Source data are provided as a Source Data file.

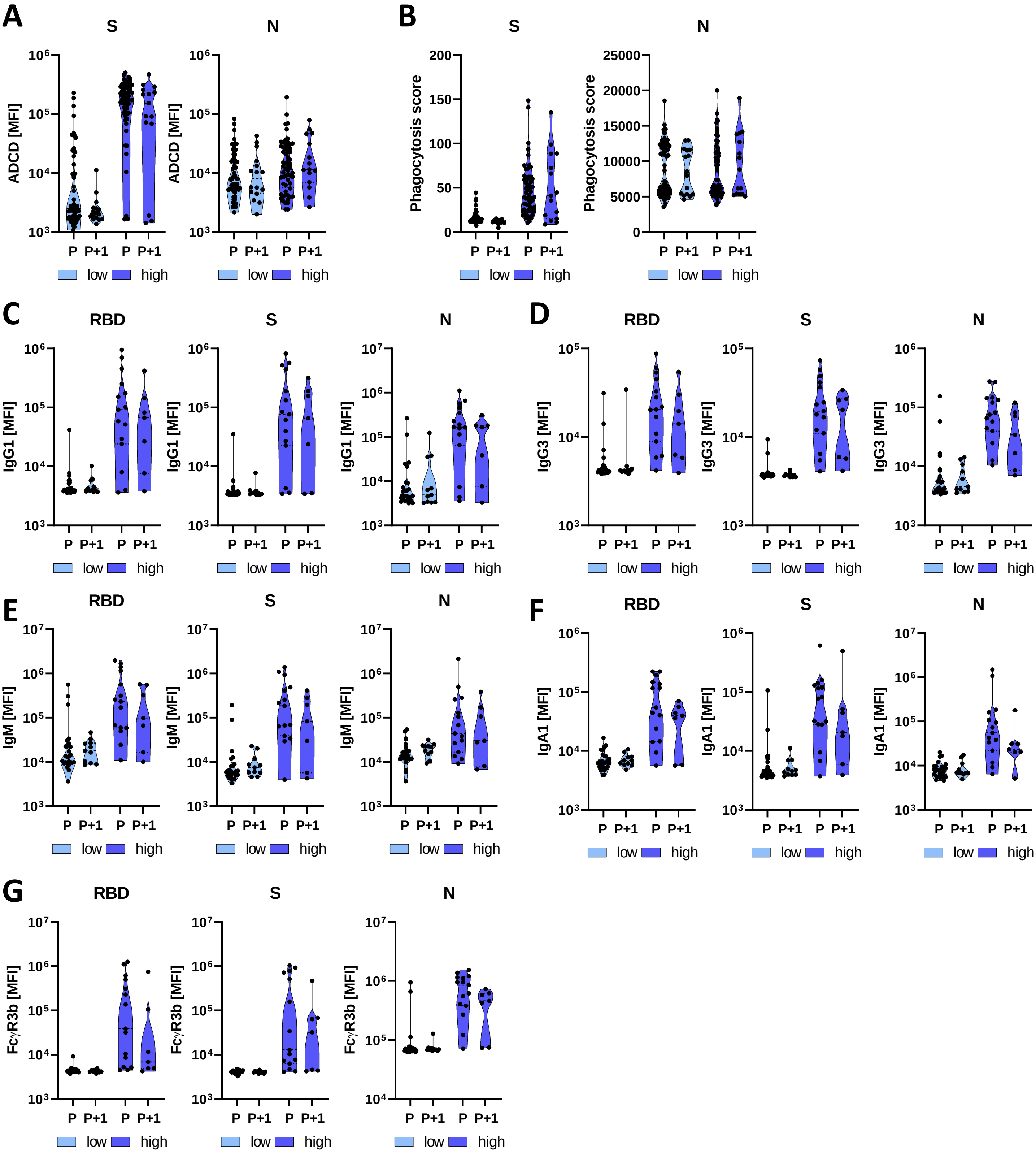

Supplementary Figure 2. SARS-CoV-2 specific antibody features in low high titer individuals at maximum observed titers or following (P+1) timepoint. (A-B) S- and N-specific ADCD (A) or ADNP (B) (max: n_{low}=60, n_{high}=60; P+1: (n_{low}=16, n_{high}=15). (C-G) RBD-, S- or N-specific IgG1 (C), IgG3 (D), IgM (E), IgA1 (F) titer, or FcγR3b binding (G) (max: n_{low}=26, n_{high}=15; P+1: (n_{low}=11, n_{high}=7). Source data are provided as a Source Data file.

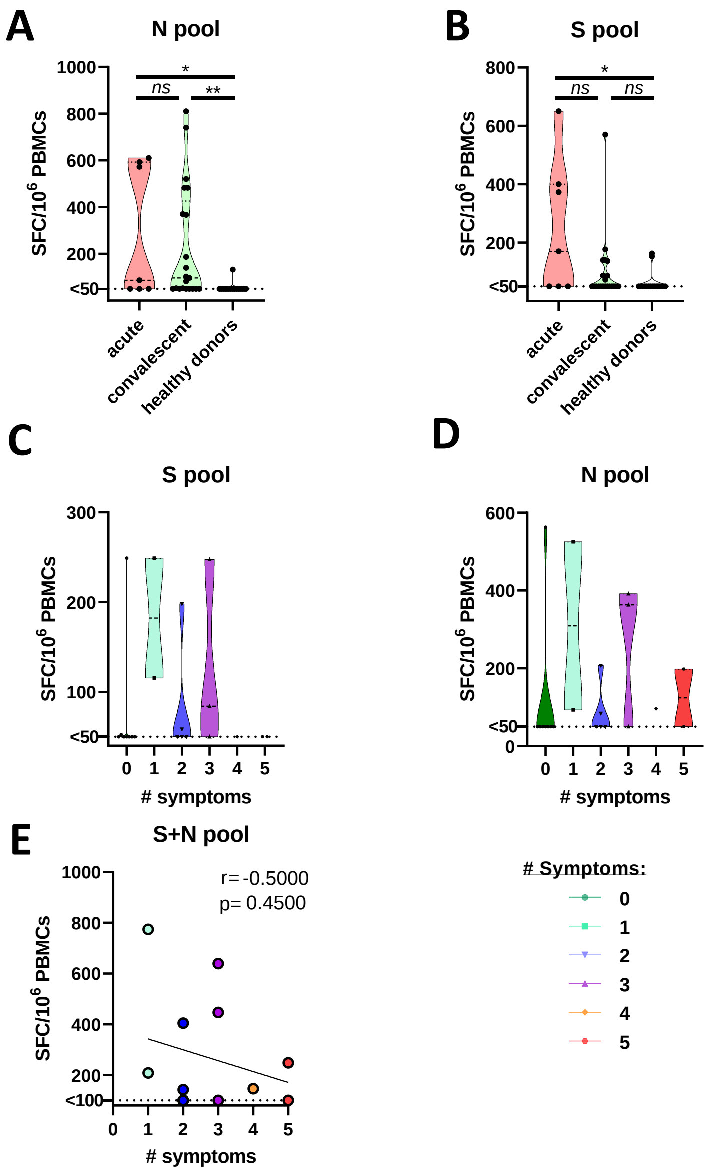

Supplementary Figure 3. Robust SARS-CoV-2 specific T cell immune responses in acutely infected and symptomatic convalescent subjects. A) and B) The violin plots show spot forming cells (SFC) of interferon-gamma (IFNγ) secreting T cells after the stimulation with N or S peptide pools across a group of acutely infected (n=7) and symptomatic convalescent (n=21) samples that were run in parallel with the low and high titer asymptomatic/mildly-infected subjects (Figure 4). Pre-pandemic blood donors served as healthy controls (n=16). C) and D) N- and S-specific T cell immune responses in Figure 4F+G were stratified by reported symptoms (values for individuals with multiple symptoms are shown for each symptom individually; LOS= loss of smell, LOT = loss of taste). No statistically significant difference was observed n_0=8, n_1=2, n_2=5, n_3=3, n_4=1, n_5=2). E) Spearman correlation of S and N pool specific T cells by number of reported symptoms. S and N pool specific SFC/10^6 PBMCs were summed up for this analysis. Statistical differences across groups in A-D were assessed with a non-parametric Kruskal-Wallis test followed by a post-hoc Dunn's correction for multiple testing: in A) *:p=0.038 , **:p=0.002; in B) *:p=0.036; ns: not significant. Source data are provided as a Source Data file.

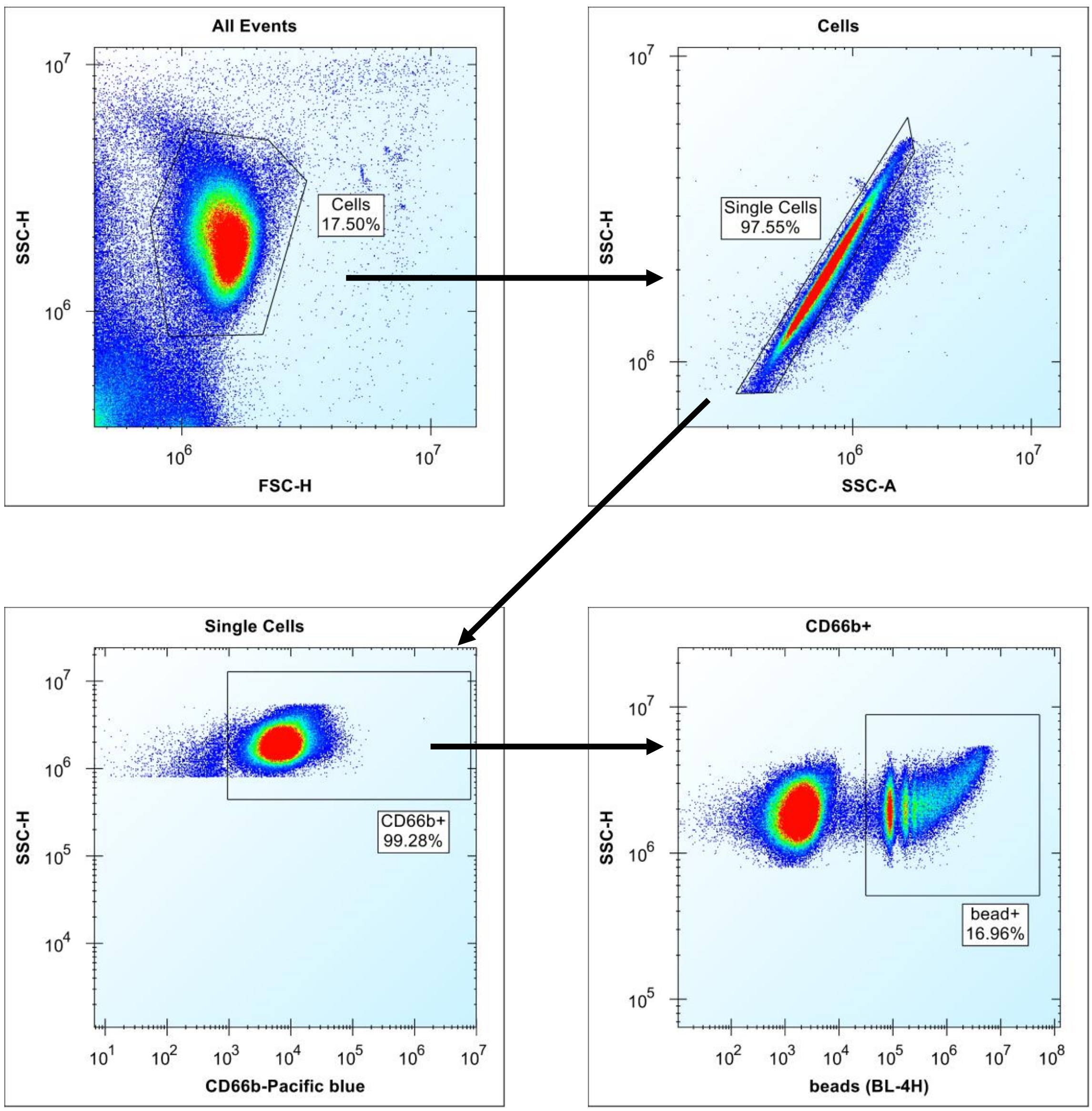

Supplementary Figure 4. Gating strategy for Antibody Dependent Neutrophil Phagocytosis (ADNP). The graphs show the gating strategy used to assess neutrophil phagocytic activity presented in Figure 3B+C, Figure 4E and Suppl. Figure 2B.
