# Supplementary material for: Discrete SARS-CoV-2 antibody titers track with functional humoral stability
Source: Nat Commun. 2021 Feb 15;12:1018. doi: 10.1038/s41467-021-21336-8 (PMC7884400; doi:10.1038/s41467-021-21336-8)
Supplement: Supplementary file 2 — Reporting Summary [file 41467_2021_21336_MOESM2_ESM.pdf]

nature research

Corresponding author(s): Gait Alter
Last updated by author(s): Jan 5, 2021

Reporting Summary

Nature Research wishes to improve the reproducibility of the work that we publish. This form provides structure for consistency and transparency in reporting. For further information on Nature Research policies, see our Editorial Policies and the Editorial Policy Checklist.

Statistics

For all statistical analyses, confirm that the following items are present in the figure legend, table legend, main text, or Methods section.

n/a	Confirmed	
☐	☒	The exact sample size (n) for each experimental group/condition, given as a discrete number and unit of measurement
☐	☒	A statement on whether measurements were taken from distinct samples or whether the same sample was measured repeatedly
☐	☒	The statistical test(s) used AND whether they are one- or two-sided<br>*Only common tests should be described solely by name; describe more complex techniques in the Methods section.*
☐	☒	A description of all covariates tested
☐	☒	A description of any assumptions or corrections, such as tests of normality and adjustment for multiple comparisons
☐	☒	A full description of the statistical parameters including central tendency (e.g. means) or other basic estimates (e.g. regression coefficient) AND variation (e.g. standard deviation) or associated estimates of uncertainty (e.g. confidence intervals)
☐	☒	For null hypothesis testing, the test statistic (e.g. F, t, r) with confidence intervals, effect sizes, degrees of freedom and P value noted<br>*Give P values as exact values whenever suitable.*
☒	☐	For Bayesian analysis, information on the choice of priors and Markov chain Monte Carlo settings
☒	☐	For hierarchical and complex designs, identification of the appropriate level for tests and full reporting of outcomes
☒	☐	Estimates of effect sizes (e.g. Cohen's d, Pearson's r), indicating how they were calculated

Our web collection on statistics for biologists contains articles on many of the points above.

Software and code

Policy information about availability of computer code

Data collection	ForeCyt® Standard Edition 8.1 was used to collect Luminex, ADNP and ADCD assay.<br>Tecan-i-control V.3.4.2 was used to collect ELISA data.
Data analysis	Microsoft Excel 365 was used to compile experimental data and patient information.<br>Violin plots and statistical analysis was performed with GraphPad Prism v.8.4.2.<br>Flower plots were generated in RStudio (v.1.3 and R v.4.0) using 'ggplot2' package (v.3.3).

For manuscripts utilizing custom algorithms or software that are central to the research but not yet described in published literature, software must be made available to editors and reviewers. We strongly encourage code deposition in a community repository (e.g. GitHub). See the Nature Research guidelines for submitting code & software for further information.

Data

Policy information about availability of data

All manuscripts must include a data availability statement. This statement should provide the following information, where applicable:
- Accession codes, unique identifiers, or web links for publicly available datasets
- A list of figures that have associated raw data
- A description of any restrictions on data availability

All relevant data is included in this manuscript. No data was stored externally. Additional protocols or raw data will be made available upon request.

Field-specific reporting

Please select the one below that is the best fit for your research. If you are not sure, read the appropriate sections before making your selection.

- [x] Life sciences
- [ ] Behavioural & social sciences
- [ ] Ecological, evolutionary & environmental sciences

For a reference copy of the document with all sections, see nature.com/documents/nr-reporting-summary-flat.pdf

Life sciences study design

All studies must disclose on these points even when the disclosure is negative.

Sample size	No sample size calculation was performed. 4300 volunteers were screened for occurrence of SARS-CoV-2 antibodies. 120 individuals with positive antibody test were included for further analysis and antibody profiling here. Based on experiences from previous studies, sample sizes are assumed to be sufficient.
Data exclusions	No data was excluded.
Replication	Seropositivity of samples was confirmed by repetition of the ELISA. All assays were run in duplicate.
Randomization	Samples from seropositive individuals were randomly distributed on 96 well plates.
Blinding	Investigators were blinded during data collection. Due to the retrospective grouping based on antibody titers, blinding of investigators was not possible with regard to titer group (high titer, low titer) during data analysis.

Reporting for specific materials, systems and methods

We require information from authors about some types of materials, experimental systems and methods used in many studies. Here, indicate whether each material, system or method listed is relevant to your study. If you are not sure if a list item applies to your research, read the appropriate section before selecting a response.

Materials & experimental systems

n/a	Involved in the study	
[ ]	[x]	Antibodies
[ ]	[x]	Eukaryotic cell lines
[x]	[ ]	Palaeontology and archaeology
[x]	[ ]	Animals and other organisms
[ ]	[x]	Human research participants
[x]	[ ]	Clinical data
[x]	[ ]	Dual use research of concern

Methods

n/a	Involved in the study	
[x]	[ ]	ChIP-seq
[ ]	[x]	Flow cytometry
[x]	[ ]	MRI-based neuroimaging

Antibodies

Antibodies used

1 .Mouse Anti-Human IgG1-PE (Southern-Biotech, #9054-09, clone:HP6001)
2. Mouse Anti-Human IgG3-PE (Southern-Biotech, #9210-09, clone:HP6050)
3. Mouse Anti-Human IgM-PE (Southern-Biotech, #9020-09, clone:SA-DA4)
4. Mouse Anti-Human IgA1-PE (Southern-Biotech, #9130-09, clone: B3506B4
5. Anti-guinea pig complement C3 goat IgG fraction (MP Biomedical, #855385. polyclonal)
6. anti-human CD66b Pacific Blue (Biolegend, #305112 ,clone G10F5)
7. anti-human CD3 (Absolute Antibody Ltd, #AB00640-2.0, clone 12F6)
8. anti human IFNg (Mabtech Inc, #3420-3-1000, clone 1-DK1)
9. anti-humna IFNg biotinylated (Mabtech Inc, #3420-6-1000, clone 7-B6-1)
10. anti-human IgG-Fc-HRP (Bethyl Labs, #A80-148P, polyclonal)
11. anti-SARS-CoV-2-RBD nonoclonal IgG1

Validation

The production and use of the anti-SARS-CoV-2-RBD antibody is described in Roy et al. SARS-CoV-2-specific ELISA development, J Immunol Methods. 2020 PMID: 32780998
All other antibodies are well established and quality controlled by the manufacturer. Additional information and references can be obtained on the company websites.

Eukaryotic cell lines

Policy information about cell lines

Cell line source(s)	HEK293T
Authentication	Commerically purchased (ATCC) and evaluated in control experiments prior to use
Mycoplasma contamination	Negative for mycoplasma
Commonly misidentified lines (See ICLAC register)	None

Human research participants

Policy information about studies involving human research participants

Population characteristics	From a total of 4300 volunteers participating in this study, 120 seropositive individuals were selected for subsequent antibody analysis. Median age of the seropositive population was 31 years (range 22 - 71 years) and 92 % were males resembling the characteristics of the parent cohort (median age: 32 years, range: 18-71 years, 84.3 % male (3582/4245 individuals with reported gender)).
Recruitment	Employees of Space Exploration Technologies Corp. (SpaceX) were invited to participate into the study. Informed consent was given before sample collection.Regular blood draws were performed and volunteers invited by email. Participants were not required to follow a specific visit schedule but decided on their own when and whether they donated sample. Previous symptoms, known exposure (e.g. household contacts) or high local incident rates may have biased a decision to donate blood (e.g. out of curiosity). However, we do not anticipate an influence on the results, since only seropositive samples were selected for further in-depth profiling .
Ethics oversight	The study protocol was approved by the Western Institutional Review Board. The use of de-identified data and biological samples was approved by the Mass General Brigham Healthcare (previously Partners Healthcare) Institutional Review Board.

Note that full information on the approval of the study protocol must also be provided in the manuscript.

Flow Cytometry

Plots

Confirm that:

- [x] The axis labels state the marker and fluorochrome used (e.g. CD4-FITC).
- [x] The axis scales are clearly visible. Include numbers along axes only for bottom left plot of group (a 'group' is an analysis of identical markers).
- [x] All plots are contour plots with outliers or pseudocolor plots.
- [x] A numerical value for number of cells or percentage (with statistics) is provided.

Methodology

Sample preparation	After phagocytosis incubation, cells were stained for CD66b surface expression and fixed with 4% para-formaldehyde thereafter. Cells were resuspended in PBS and stored at 4C protected from light for up to 24h, if immediate flow cytometric analysis was not immediately possible.
Instrument	IntelliCyt® iQue Screener PLUS
Software	ForeCyt® Standard Edition 8.1 was used to collect and analyze the data.
Cell population abundance	Neutrophil purity was assessed by CD66b expression in the granulocyte gate. The fraction of CD66b positive granulocytes was >95% for all donors.
Gating strategy	All events were gated for granulocutes using FSC-H and SSC-H and single cells subsequently selected using SSC-A and SSC-H. Neutrophils were selected for CD66b expression. Phagocytic cells identified in the BL4-H channel.

- [x] Tick this box to confirm that a figure exemplifying the gating strategy is provided in the Supplementary Information.
